# Hexa­kis­(4-acetyl­pyridinium) octa­deca­chloridotetra­anti­monate(III)

**DOI:** 10.1107/S160053681002057X

**Published:** 2010-06-05

**Authors:** Xue-qun Fu

**Affiliations:** a Ordered Matter Science Research Center, Southeast University, Nanjing 210096, People’s Republic of China

## Abstract

The title compound, (C_7_H_8_NO)_6_[Sb_4_Cl_18_], contains centrosymmetric hexa­anions built up from four vertex-sharing alternating SbCl_5_ square-based pyramids and highly distorted SbCl_6_ octa­hedra when long (<3.2 Å) ‘secondary’ Sb—Cl inter­actions are taken into account. The inter-polyhedral Sb—Cl bonds define a square-shape. In the crystal, the components are linked by N—H⋯Cl, C—H⋯Cl and C—H⋯O hydrogen bonds, generating a three-dimensional network.

## Related literature

For general background to phase transitions in coordination networks, see: Li *et al.* (2008 ▶); Zhang *et al.* (2009 ▶). For crystal structures containing the 4-acetyl­pyridinium cation, see: Fu (2009*a* ▶,*b* ▶); Majerz *et al.* (1991 ▶); Pang *et al.* (1994 ▶); Steffen & Palenik (1977 ▶).
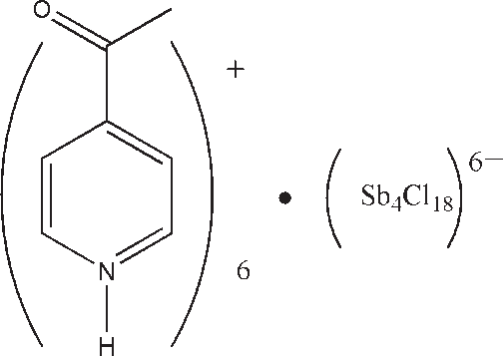


## Experimental

### 

#### Crystal data

(C_7_H_8_NO)_6_[Sb_4_Cl_18_]
*M* *_r_* = 1857.96
Triclinic, 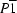

*a* = 9.0589 (18) Å
*b* = 13.838 (3) Å
*c* = 15.128 (3) Å
α = 108.29 (3)°
β = 98.00 (3)°
γ = 107.10 (3)°
*V* = 1664.1 (6) Å^3^
*Z* = 1
Mo *K*α radiation
μ = 2.37 mm^−1^
*T* = 298 K
0.40 × 0.30 × 0.20 mm

#### Data collection

Rigaku SCXmini diffractometer
Absorption correction: multi-scan (*CrystalClear*; Rigaku, 2005 ▶) *T*_min_ = 0.430, *T*_max_ = 0.622
17638 measured reflections
7613 independent reflections
6371 reflections with *I* > 2σ(*I*)
*R*_int_ = 0.028

#### Refinement

*R*[*F* ^2^ > 2σ(*F* ^2^)] = 0.030
*wR*(*F* ^2^) = 0.070
*S* = 1.04
7613 reflections
343 parameters
H-atom parameters constrained
Δρ_max_ = 0.39 e Å^−3^
Δρ_min_ = −0.38 e Å^−3^

### 

Data collection: *CrystalClear* (Rigaku, 2005 ▶); cell refinement: *CrystalClear*; data reduction: *CrystalClear*; program(s) used to solve structure: *SHELXS97* (Sheldrick, 2008 ▶); program(s) used to refine structure: *SHELXL97* (Sheldrick, 2008 ▶); molecular graphics: *SHELXTL* (Sheldrick, 2008 ▶); software used to prepare material for publication: *SHELXL97*.

## Supplementary Material

Crystal structure: contains datablocks I, global. DOI: 10.1107/S160053681002057X/hb5440sup1.cif

Structure factors: contains datablocks I. DOI: 10.1107/S160053681002057X/hb5440Isup2.hkl

Additional supplementary materials:  crystallographic information; 3D view; checkCIF report

## Figures and Tables

**Table 1:** Selected bond lengths (Å)

Sb1—Cl4	2.4036 (9)
Sb1—Cl3	2.4107 (10)
Sb1—Cl2	2.4113 (14)
Sb1—Cl1	2.9359 (12)
Sb1—Cl5	3.0214 (12)
Sb1—Cl6^i^	3.1275 (12)
Sb2—Cl7	2.3516 (12)
Sb2—Cl8	2.4459 (10)
Sb2—Cl9	2.4498 (10)
Sb2—Cl5	2.8352 (11)
Sb2—Cl6	2.8937 (11)

Symmetry code: (i) 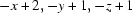.

**Table 2:** Hydrogen-bond geometry (Å, °)

*D*—H⋯*A*	*D*—H	H⋯*A*	*D*⋯*A*	*D*—H⋯*A*
N2—H2*A*⋯Cl6	0.86	2.30	3.148 (3)	170
N1—H1*D*⋯Cl1^ii^	0.86	2.20	3.056 (3)	174
N3—H3*A*⋯Cl5^iii^	0.86	2.35	3.198 (3)	168
C1—H1*A*⋯O2^iv^	0.96	2.60	3.506 (5)	158
C5—H5*A*⋯Cl8^v^	0.93	2.78	3.585 (4)	146
C13—H13*A*⋯Cl1^i^	0.93	2.76	3.661 (4)	162
C19—H19*A*⋯Cl7^iii^	0.93	2.67	3.449 (4)	141
C21—H21*A*⋯O1^iii^	0.93	2.42	3.349 (4)	177

Symmetry codes: (i) 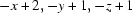; (ii) 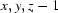; (iii) 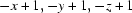; (iv) 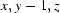; (v) 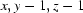.

**Fig. 1.**
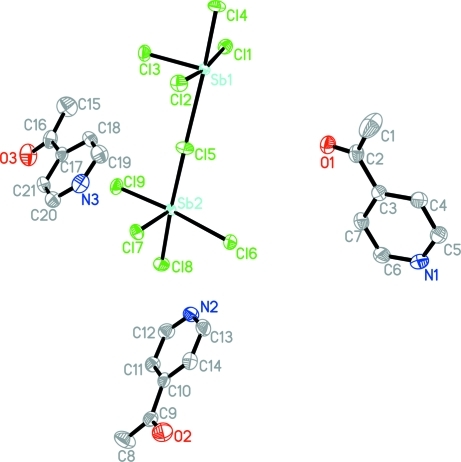
The molecular structure of (I) with displacement ellipsoids drawn at the 30% probability level (all H atoms have been omitted for clarity). Unlabelled atoms are generated by the symmetry operation (2–x, 1–y, 1–z).

**Fig. 2.**
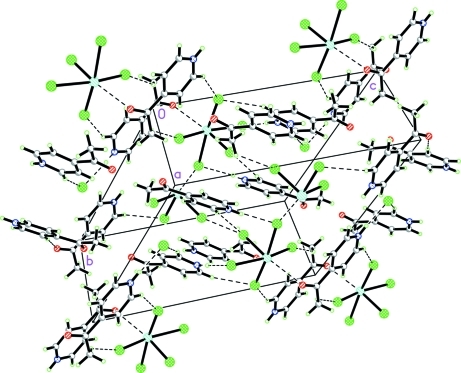
A view of the packing of (I), stacking along the a axis. Dashed lines indicate hydrogen bonds.

## supplementary crystallographic information

### Comment

As a continuation of our study of phase transition materials, including organic ligands (Li *et al.*, 2008), metal-organic coordination compounds (Zhang *et al.*, 2009), organic-inorganic hybrids, we studied the dielectric properties of the title compound, unfortunately, there was no distinct anomaly observed from 93 K to 400 K, (m.p. 421–423 K), suggesting that this compound should be not a real ferroelectrics or there may be no distinct phase transition occurred within the measured temperature range. In this article, the crystal structure of (I) has been presented.

4-Acetylpyridine may be used as a ligand in coordination compounds *e.g.* with Zn (Steffen & Palenik, 1977) or Ni (Pang *et al.*, 1994). The crystal structures of 4-acetylpyridine together with inorganic acids are also known *e.g.* with sulfuric acid (Fu, 2009b) and perchloric acid (Fu, 2009a).

The cell unit of the title compound is made up of six almost planar protonated 4-acetylpyridinium cations and a [Sb_4_Cl_18_]^6-^ anion (Fig. 1.).In the coordinate anion of [Sb_4_Cl_18_]^6-^, antimony(III) atoms have two kinds of coordination pattern. Sb^3+^(2) coordinated with five Cl ions construct a distorted tetragonal pyramidal structure, composing two briding and three terminal Cl atoms. There are Cl—Sb secondary bonds by the linkage between the Sb^3+^(1)···Cl5 and Sb^3+^(1)···Cl6, with the bond lengths of these secondary bonds 3.0210 (11)Å and 3.1280 (11) Å, respectively, compared to the normal coordination bonds of Sb—Cl 2.3516 (12)Å to 2.8937 (11) Å. Owing to these secondary bonds, the coordination number of the central ion Sb^3+^(1) increases to six, and it adopts a distorted octahedral geometry.

The tridimensional network arrangement in the crystal structure of (I) is mainly determined by relatively strong and directional hydrogen bonds (Table. 1),

### Experimental

2.28 g (10 mmol) SbCl_3_ was firstly dissolved in 10 ml 1:1 HCl solution, to which 2.42 g (20 mmol) 4-acetylpyridine ethanol solution was then added under stirring. Hydrochloric acid was added until the precipitated substrates disappeared. The acid solution was allowed to slowly evaporate at room temperature until colorless prisms of (I) were grown.

### Refinement

Positional parameters of all the H atoms were calculated geometrically and were allowed to ride on the C and N atoms to which they are bonded, with *U*_iso_(H) = 1.2*U*_eq_(C),

*U*_iso_(H) = 1.2*U*_eq_(N).

### Crystal data

**Table:**

(C_7_H_8_NO)_6_[Sb_4_Cl_18_]	*Z* = 1
*M**_r_* = 1857.96	*F*(000) = 900
Triclinic, *P*1	*D*_x_ = 1.854 Mg m^−^^3^
Hall symbol: -P 1	Mo *K*α radiation, λ = 0.71073 Å
*a* = 9.0589 (18) Å	Cell parameters from 8056 reflections
*b* = 13.838 (3) Å	θ = 3.1–27.7°
*c* = 15.128 (3) Å	µ = 2.37 mm^−^^1^
α = 108.29 (3)°	*T* = 298 K
β = 98.00 (3)°	Prism, colourless
γ = 107.10 (3)°	0.40 × 0.30 × 0.20 mm
*V* = 1664.1 (6) Å^3^	

### Data collection

**Table:**

Rigaku SCXmini diffractometer	7613 independent reflections
Radiation source: fine-focus sealed tube	6371 reflections with *I* > 2σ(*I*)
graphite	*R*_int_ = 0.028
Detector resolution: 13.6612 pixels mm^-1^	θ_max_ = 27.5°, θ_min_ = 3.1°
ω scans	*h* = −11→11
Absorption correction: multi-scan (*CrystalClear*; Rigaku, 2005)	*k* = −17→17
*T*_min_ = 0.430, *T*_max_ = 0.622	*l* = −19→19
17638 measured reflections	

### Refinement

**Table:**

Refinement on *F*^2^	Primary atom site location: structure-invariant direct methods
Least-squares matrix: full	Secondary atom site location: difference Fourier map
*R*[*F*^2^ > 2σ(*F*^2^)] = 0.030	Hydrogen site location: inferred from neighbouring sites
*wR*(*F*^2^) = 0.070	H-atom parameters constrained
*S* = 1.04	*w* = 1/[σ^2^(*F*_o_^2^) + (0.0307*P*)^2^ + 0.5066*P*] where *P* = (*F*_o_^2^ + 2*F*_c_^2^)/3
7613 reflections	(Δ/σ)_max_ = 0.001
343 parameters	Δρ_max_ = 0.39 e Å^−^^3^
0 restraints	Δρ_min_ = −0.38 e Å^−^^3^

### Special details

**Table:**

Geometry. All e.s.d.'s (except the e.s.d. in the dihedral angle between two l.s. planes) are estimated using the full covariance matrix. The cell e.s.d.'s are taken into account individually in the estimation of e.s.d.'s in distances, angles and torsion angles; correlations between e.s.d.'s in cell parameters are only used when they are defined by crystal symmetry. An approximate (isotropic) treatment of cell e.s.d.'s is used for estimating e.s.d.'s involving l.s. planes.
Refinement. Refinement of *F*^2^ against ALL reflections. The weighted *R*-factor *wR* and goodness of fit *S* are based on *F*^2^, conventional *R*-factors *R* are based on *F*, with *F* set to zero for negative *F*^2^. The threshold expression of *F*^2^ > σ(*F*^2^) is used only for calculating *R*-factors(gt) *etc*. and is not relevant to the choice of reflections for refinement. *R*-factors based on *F*^2^ are statistically about twice as large as those based on *F*, and *R*- factors based on ALL data will be even larger.

### Fractional atomic coordinates and isotropic or equivalent isotropic displacement parameters (Å^2^)

**Table:**

	*x*	*y*	*z*	*U*_iso_*/*U*_eq_	
O1	0.7595 (3)	0.1417 (2)	0.15768 (17)	0.0544 (6)	
N1	0.9897 (4)	0.2973 (3)	−0.0627 (2)	0.0573 (8)	
H1D	1.0377	0.3389	−0.0898	0.069*	
C3	0.8404 (4)	0.1681 (3)	0.0233 (2)	0.0408 (7)	
C6	0.9436 (5)	0.3405 (3)	0.0152 (3)	0.0574 (10)	
H6A	0.9616	0.4147	0.0386	0.069*	
C4	0.8871 (4)	0.1248 (3)	−0.0588 (2)	0.0490 (8)	
H4A	0.8660	0.0503	−0.0855	0.059*	
C7	0.8713 (4)	0.2783 (3)	0.0610 (3)	0.0525 (9)	
H7A	0.8428	0.3094	0.1169	0.063*	
C5	0.9634 (5)	0.1912 (3)	−0.0998 (2)	0.0557 (9)	
H5A	0.9976	0.1629	−0.1540	0.067*	
C2	0.7623 (4)	0.0986 (3)	0.0748 (3)	0.0497 (8)	
Sb1	0.86744 (2)	0.350428 (15)	0.674838 (14)	0.03137 (6)	
Cl4	0.83619 (12)	0.17158 (6)	0.67419 (6)	0.0519 (2)	
Cl3	0.74694 (11)	0.39130 (8)	0.80769 (6)	0.0528 (2)	
Cl2	0.61006 (10)	0.27520 (7)	0.56102 (6)	0.0543 (2)	
C1	0.6945 (7)	−0.0208 (3)	0.0234 (4)	0.110 (2)	
H1A	0.6487	−0.0545	0.0648	0.165*	
H1B	0.7775	−0.0467	0.0054	0.165*	
H1C	0.6133	−0.0390	−0.0333	0.165*	
Cl1	1.15940 (10)	0.43033 (7)	0.82937 (7)	0.0529 (2)	
Sb2	0.93951 (2)	0.785682 (15)	0.693125 (14)	0.03208 (6)	
Cl7	0.67862 (9)	0.73910 (7)	0.60241 (6)	0.0474 (2)	
Cl9	0.83859 (11)	0.82021 (8)	0.83764 (6)	0.0544 (2)	
Cl8	0.99930 (12)	0.97530 (7)	0.70831 (7)	0.0595 (2)	
Cl5	0.86213 (12)	0.56019 (7)	0.65539 (7)	0.0577 (2)	
Cl6	1.00732 (11)	0.73440 (8)	0.50601 (6)	0.0557 (2)	
C10	0.6128 (4)	0.9155 (3)	0.3188 (2)	0.0436 (8)	
C14	0.6375 (4)	0.8208 (3)	0.2737 (3)	0.0525 (9)	
H14A	0.5913	0.7814	0.2082	0.063*	
N2	0.7927 (4)	0.8408 (3)	0.4180 (3)	0.0584 (8)	
H2A	0.8511	0.8172	0.4493	0.070*	
C12	0.7702 (5)	0.9308 (3)	0.4648 (3)	0.0595 (10)	
H12A	0.8150	0.9669	0.5308	0.071*	
C11	0.6807 (4)	0.9711 (3)	0.4161 (3)	0.0512 (9)	
H11A	0.6659	1.0355	0.4484	0.061*	
C13	0.7289 (5)	0.7851 (3)	0.3245 (3)	0.0610 (10)	
H13A	0.7471	0.7216	0.2939	0.073*	
O2	0.4633 (4)	0.9034 (3)	0.1749 (2)	0.0794 (9)	
C9	0.5172 (4)	0.9568 (3)	0.2594 (3)	0.0537 (9)	
C8	0.4958 (6)	1.0602 (4)	0.3054 (3)	0.0865 (15)	
H8A	0.4326	1.0748	0.2582	0.130*	
H8B	0.5982	1.1180	0.3327	0.130*	
H8C	0.4424	1.0555	0.3553	0.130*	
C18	0.4716 (4)	0.5259 (3)	0.7037 (3)	0.0540 (9)	
H18A	0.5403	0.4988	0.7313	0.065*	
N3	0.3089 (4)	0.5204 (3)	0.5680 (2)	0.0584 (8)	
H3A	0.2674	0.4905	0.5069	0.070*	
C17	0.4345 (4)	0.6097 (2)	0.7603 (2)	0.0418 (7)	
C16	0.4972 (4)	0.6600 (3)	0.8688 (3)	0.0587 (10)	
C21	0.3347 (4)	0.6486 (3)	0.7166 (3)	0.0537 (9)	
H21A	0.3100	0.7066	0.7534	0.064*	
C15	0.5935 (6)	0.6125 (4)	0.9162 (3)	0.0897 (15)	
H15A	0.6250	0.6526	0.9845	0.135*	
H15B	0.6870	0.6161	0.8924	0.135*	
H15C	0.5314	0.5377	0.9027	0.135*	
C20	0.2716 (5)	0.6023 (3)	0.6191 (3)	0.0588 (10)	
H20A	0.2035	0.6280	0.5892	0.071*	
O3	0.4681 (4)	0.7375 (2)	0.9125 (2)	0.0803 (9)	
C19	0.4076 (5)	0.4828 (3)	0.6073 (3)	0.0642 (11)	
H19A	0.4332	0.4264	0.5686	0.077*	

### Atomic displacement parameters (Å^2^)

**Table:**

	*U*^11^	*U*^22^	*U*^33^	*U*^12^	*U*^13^	*U*^23^
O1	0.0600 (16)	0.0584 (15)	0.0478 (14)	0.0245 (12)	0.0219 (12)	0.0173 (12)
N1	0.068 (2)	0.063 (2)	0.0528 (19)	0.0261 (16)	0.0166 (16)	0.0331 (17)
C3	0.0408 (17)	0.0425 (18)	0.0348 (17)	0.0151 (14)	0.0048 (13)	0.0109 (15)
C6	0.075 (3)	0.046 (2)	0.058 (2)	0.0281 (19)	0.023 (2)	0.0205 (19)
C4	0.065 (2)	0.0417 (19)	0.0358 (18)	0.0204 (17)	0.0090 (16)	0.0096 (15)
C7	0.060 (2)	0.049 (2)	0.050 (2)	0.0259 (17)	0.0191 (17)	0.0118 (17)
C5	0.076 (3)	0.060 (2)	0.0344 (18)	0.031 (2)	0.0144 (18)	0.0157 (18)
C2	0.050 (2)	0.047 (2)	0.048 (2)	0.0174 (16)	0.0158 (16)	0.0107 (17)
Sb1	0.03433 (11)	0.03025 (11)	0.03262 (11)	0.01447 (8)	0.00818 (8)	0.01327 (9)
Cl4	0.0760 (6)	0.0343 (4)	0.0502 (5)	0.0230 (4)	0.0131 (4)	0.0206 (4)
Cl3	0.0535 (5)	0.0654 (6)	0.0479 (5)	0.0294 (4)	0.0235 (4)	0.0201 (4)
Cl2	0.0417 (4)	0.0604 (5)	0.0524 (5)	0.0143 (4)	−0.0025 (4)	0.0199 (4)
C1	0.156 (5)	0.050 (3)	0.100 (4)	0.001 (3)	0.076 (4)	0.012 (3)
Cl1	0.0454 (5)	0.0531 (5)	0.0556 (5)	0.0120 (4)	0.0120 (4)	0.0201 (4)
Sb2	0.03311 (11)	0.03102 (11)	0.03265 (11)	0.01310 (8)	0.00899 (8)	0.01088 (9)
Cl7	0.0387 (4)	0.0537 (5)	0.0463 (5)	0.0207 (4)	0.0039 (3)	0.0133 (4)
Cl9	0.0555 (5)	0.0718 (6)	0.0403 (4)	0.0266 (4)	0.0207 (4)	0.0195 (4)
Cl8	0.0743 (6)	0.0327 (4)	0.0724 (6)	0.0173 (4)	0.0249 (5)	0.0198 (4)
Cl5	0.0680 (6)	0.0411 (5)	0.0661 (6)	0.0207 (4)	0.0095 (5)	0.0252 (4)
Cl6	0.0632 (6)	0.0692 (6)	0.0527 (5)	0.0359 (5)	0.0274 (4)	0.0295 (5)
C10	0.0378 (17)	0.0436 (18)	0.050 (2)	0.0115 (14)	0.0189 (15)	0.0174 (16)
C14	0.049 (2)	0.045 (2)	0.054 (2)	0.0113 (16)	0.0148 (17)	0.0098 (17)
N2	0.0512 (18)	0.062 (2)	0.074 (2)	0.0242 (16)	0.0188 (17)	0.0363 (19)
C12	0.060 (2)	0.066 (3)	0.051 (2)	0.021 (2)	0.0104 (19)	0.024 (2)
C11	0.058 (2)	0.0444 (19)	0.048 (2)	0.0174 (17)	0.0160 (17)	0.0134 (17)
C13	0.062 (2)	0.045 (2)	0.084 (3)	0.0245 (19)	0.032 (2)	0.025 (2)
O2	0.086 (2)	0.097 (2)	0.0539 (18)	0.0350 (18)	0.0098 (16)	0.0284 (18)
C9	0.049 (2)	0.064 (2)	0.054 (2)	0.0180 (18)	0.0191 (18)	0.029 (2)
C8	0.115 (4)	0.073 (3)	0.086 (3)	0.058 (3)	0.019 (3)	0.029 (3)
C18	0.052 (2)	0.050 (2)	0.063 (2)	0.0253 (17)	0.0139 (18)	0.0182 (19)
N3	0.0535 (19)	0.059 (2)	0.0435 (17)	0.0063 (15)	0.0064 (14)	0.0091 (15)
C17	0.0345 (16)	0.0369 (17)	0.0491 (19)	0.0089 (13)	0.0141 (14)	0.0118 (15)
C16	0.043 (2)	0.062 (2)	0.054 (2)	0.0087 (18)	0.0123 (17)	0.011 (2)
C21	0.057 (2)	0.056 (2)	0.060 (2)	0.0328 (18)	0.0253 (19)	0.0211 (19)
C15	0.084 (3)	0.105 (4)	0.068 (3)	0.033 (3)	−0.005 (3)	0.028 (3)
C20	0.055 (2)	0.072 (3)	0.062 (3)	0.030 (2)	0.0186 (19)	0.034 (2)
O3	0.082 (2)	0.0704 (19)	0.0608 (18)	0.0244 (16)	0.0179 (15)	−0.0077 (15)
C19	0.081 (3)	0.046 (2)	0.057 (2)	0.026 (2)	0.020 (2)	0.0038 (19)

### Geometric parameters (Å, °)

**Table:**

O1—C2	1.215 (4)	C14—C13	1.350 (5)
N1—C6	1.327 (4)	C14—H14A	0.9300
N1—C5	1.329 (5)	N2—C12	1.316 (5)
N1—H1D	0.8600	N2—C13	1.325 (5)
C3—C7	1.374 (4)	N2—H2A	0.8600
C3—C4	1.381 (4)	C12—C11	1.367 (5)
C3—C2	1.502 (5)	C12—H12A	0.9300
C6—C7	1.348 (5)	C11—H11A	0.9300
C6—H6A	0.9300	C13—H13A	0.9300
C4—C5	1.345 (5)	O2—C9	1.200 (4)
C4—H4A	0.9300	C9—C8	1.464 (5)
C7—H7A	0.9300	C8—H8A	0.9600
C5—H5A	0.9300	C8—H8B	0.9600
C2—C1	1.476 (5)	C8—H8C	0.9600
C1—H1A	0.9600	C18—C19	1.354 (5)
C1—H1B	0.9600	C18—C17	1.370 (4)
C1—H1C	0.9600	C18—H18A	0.9300
Sb1—Cl4	2.4036 (9)	N3—C20	1.320 (5)
Sb1—Cl3	2.4107 (10)	N3—C19	1.320 (5)
Sb1—Cl2	2.4113 (14)	N3—H3A	0.8600
Sb1—Cl1	2.9359 (12)	C17—C21	1.374 (5)
Sb1—Cl5	3.0214 (12)	C17—C16	1.514 (5)
Sb1—Cl6^i^	3.1275 (12)	C16—O3	1.194 (4)
Sb2—Cl7	2.3516 (12)	C16—C15	1.473 (6)
Sb2—Cl8	2.4459 (10)	C21—C20	1.367 (5)
Sb2—Cl9	2.4498 (10)	C21—H21A	0.9300
Sb2—Cl5	2.8352 (11)	C15—H15A	0.9600
Sb2—Cl6	2.8937 (11)	C15—H15B	0.9600
C10—C14	1.373 (4)	C15—H15C	0.9600
C10—C11	1.378 (5)	C20—H20A	0.9300
C10—C9	1.511 (5)	C19—H19A	0.9300
			
C6—N1—C5	121.5 (3)	C12—N2—H2A	118.7
C6—N1—H1D	119.2	C13—N2—H2A	118.7
C5—N1—H1D	119.2	N2—C12—C11	119.7 (4)
C7—C3—C4	119.3 (3)	N2—C12—H12A	120.1
C7—C3—C2	118.9 (3)	C11—C12—H12A	120.1
C4—C3—C2	121.8 (3)	C12—C11—C10	119.3 (3)
N1—C6—C7	121.0 (3)	C12—C11—H11A	120.3
N1—C6—H6A	119.5	C10—C11—H11A	120.3
C7—C6—H6A	119.5	N2—C13—C14	119.8 (3)
C5—C4—C3	119.5 (3)	N2—C13—H13A	120.1
C5—C4—H4A	120.2	C14—C13—H13A	120.1
C3—C4—H4A	120.2	O2—C9—C8	122.4 (4)
C6—C7—C3	118.6 (3)	O2—C9—C10	117.8 (4)
C6—C7—H7A	120.7	C8—C9—C10	119.7 (3)
C3—C7—H7A	120.7	C9—C8—H8A	109.5
N1—C5—C4	120.0 (3)	C9—C8—H8B	109.5
N1—C5—H5A	120.0	H8A—C8—H8B	109.5
C4—C5—H5A	120.0	C9—C8—H8C	109.5
O1—C2—C1	121.9 (4)	H8A—C8—H8C	109.5
O1—C2—C3	119.5 (3)	H8B—C8—H8C	109.5
C1—C2—C3	118.6 (3)	C19—C18—C17	119.5 (4)
Cl4—Sb1—Cl3	92.15 (4)	C19—C18—H18A	120.2
Cl4—Sb1—Cl2	89.42 (5)	C17—C18—H18A	120.2
Cl3—Sb1—Cl2	90.96 (4)	C20—N3—C19	122.4 (3)
C2—C1—H1A	109.5	C20—N3—H3A	118.8
C2—C1—H1B	109.5	C19—N3—H3A	118.8
H1A—C1—H1B	109.5	C18—C17—C21	118.4 (3)
C2—C1—H1C	109.5	C18—C17—C16	122.7 (3)
H1A—C1—H1C	109.5	C21—C17—C16	118.8 (3)
H1B—C1—H1C	109.5	O3—C16—C15	122.5 (4)
Cl7—Sb2—Cl8	90.05 (5)	O3—C16—C17	118.7 (4)
Cl7—Sb2—Cl9	87.97 (4)	C15—C16—C17	118.8 (4)
Cl8—Sb2—Cl9	90.76 (4)	C20—C21—C17	120.2 (3)
Cl7—Sb2—Cl5	86.83 (5)	C20—C21—H21A	119.9
Cl8—Sb2—Cl5	174.29 (3)	C17—C21—H21A	119.9
Cl9—Sb2—Cl5	93.90 (4)	C16—C15—H15A	109.5
Cl7—Sb2—Cl6	83.07 (4)	C16—C15—H15B	109.5
Cl8—Sb2—Cl6	89.72 (4)	H15A—C15—H15B	109.5
Cl9—Sb2—Cl6	171.03 (3)	C16—C15—H15C	109.5
Cl5—Sb2—Cl6	85.17 (4)	H15A—C15—H15C	109.5
C14—C10—C11	118.7 (3)	H15B—C15—H15C	109.5
C14—C10—C9	118.9 (3)	N3—C20—C21	119.0 (4)
C11—C10—C9	122.4 (3)	N3—C20—H20A	120.5
C13—C14—C10	119.9 (4)	C21—C20—H20A	120.5
C13—C14—H14A	120.0	N3—C19—C18	120.4 (3)
C10—C14—H14A	120.0	N3—C19—H19A	119.8
C12—N2—C13	122.5 (3)	C18—C19—H19A	119.8
			
C5—N1—C6—C7	1.5 (6)	C12—N2—C13—C14	0.3 (6)
C7—C3—C4—C5	0.7 (5)	C10—C14—C13—N2	0.9 (6)
C2—C3—C4—C5	−176.9 (3)	C14—C10—C9—O2	1.7 (5)
N1—C6—C7—C3	−2.4 (6)	C11—C10—C9—O2	179.5 (3)
C4—C3—C7—C6	1.3 (5)	C14—C10—C9—C8	−177.2 (4)
C2—C3—C7—C6	179.0 (3)	C11—C10—C9—C8	0.6 (5)
C6—N1—C5—C4	0.6 (6)	C19—C18—C17—C21	−1.1 (5)
C3—C4—C5—N1	−1.7 (6)	C19—C18—C17—C16	178.2 (4)
C7—C3—C2—O1	−16.1 (5)	C18—C17—C16—O3	175.1 (4)
C4—C3—C2—O1	161.5 (3)	C21—C17—C16—O3	−5.6 (5)
C7—C3—C2—C1	165.5 (4)	C18—C17—C16—C15	−4.1 (5)
C4—C3—C2—C1	−16.9 (5)	C21—C17—C16—C15	175.1 (4)
C11—C10—C14—C13	−1.0 (5)	C18—C17—C21—C20	1.6 (5)
C9—C10—C14—C13	176.9 (3)	C16—C17—C21—C20	−177.7 (3)
C13—N2—C12—C11	−1.4 (6)	C19—N3—C20—C21	−1.3 (6)
N2—C12—C11—C10	1.2 (6)	C17—C21—C20—N3	−0.4 (6)
C14—C10—C11—C12	0.0 (5)	C20—N3—C19—C18	1.9 (6)
C9—C10—C11—C12	−177.8 (3)	C17—C18—C19—N3	−0.6 (6)

Symmetry codes: (i) −*x*+2, −*y*+1, −*z*+1.

### Hydrogen-bond geometry (Å, °)

**Table:**

*D*—H···*A*	*D*—H	H···*A*	*D*···*A*	*D*—H···*A*
N2—H2A···Cl6	0.86	2.30	3.148 (3)	170
N1—H1D···Cl1^ii^	0.86	2.20	3.056 (3)	174
N3—H3A···Cl5^iii^	0.86	2.35	3.198 (3)	168
C1—H1A···O2^iv^	0.96	2.60	3.506 (5)	158
C5—H5A···Cl8^v^	0.93	2.78	3.585 (4)	146
C13—H13A···Cl1^i^	0.93	2.76	3.661 (4)	162
C19—H19A···Cl7^iii^	0.93	2.67	3.449 (4)	141
C21—H21A···O1^iii^	0.93	2.42	3.349 (4)	177

Symmetry codes: (ii) *x*, *y*, *z*−1; (iii) −*x*+1, −*y*+1, −*z*+1; (iv) *x*, *y*−1, *z*; (v) *x*, *y*−1, *z*−1; (i) −*x*+2, −*y*+1, −*z*+1.
